# The Experience of People With Dementia in Accessing and Engaging in Talking Therapies for Mental Health Difficulties: A Systematic Review and Thematic Meta-Synthesis

**DOI:** 10.1177/14713012251408694

**Published:** 2025-12-26

**Authors:** David Zammitt, Caroline Fearn, Amber John, Madalena Lykourgos, Suman Kurana, Ollie Hayes, Joshua Stott

**Affiliations:** 1 Department of Clinical, Educational and Health Psychology, 4919 University College London, London, UK

**Keywords:** dementia, talking therapies, experiences, engagement, access, systematic review, thematic meta-synthesis

## Abstract

People with dementia often experience psychological distress, and may experience difficulty in navigating changes to their cognition, identity and quality of life, yet experience challenges in accessing and engaging in talking therapies. This study aims to conduct a systematic review and thematic meta-synthesis to explore the experiences of people with dementia in accessing talking therapies by incorporating the perspectives of people with dementia, caregivers and dementia care professionals. We searched PsychINFO, CINAHL and Web of Science in March 2024 and included empirical peer reviewed studies that had qualitative data focussing on accessing, delivering or participating in talking therapies, from the perspective or people with dementia, their caregivers, or professionals. Only English language studies were included. 15 studies were included. Across the studies, qualitative data was collected from 88 people with dementia, 96 caregivers/family members/supportive others, 38 professionals and seven community stakeholders. Quality assessment was conducted using a revised version of the Critical Appraisal Skills Programme checklist for qualitative research. Talking therapies can help people with dementia to address, process, and accept difficult life situations, whilst also improving their knowledge on dementia and positively impacting their relationships with significant others. However, to maximise benefits, several adjustments are necessary, including, adjusting the length of therapy sessions, increased therapy reminders and follow-up, tailored communication, use of group sessions and improving staff’s dementia knowledge. Additionally, the use of dementia-specific tools, alongside outcome measures that measure broader wellbeing constructs are recommended. Involving people with dementia, caregivers and dementia professionals in therapy planning and delivery can also improve outcomes. Talking therapies for people with dementia are effective, though adaptations to address their unique support needs are essential. Several studies explored multicomponent interventions, making it difficult to ascertain which specific elements of psychological intervention were most effective. Additionally, individuals from ethnically diverse backgrounds were consistently underrepresented across the studies.

## Introduction

Dementia is characterised by a significant and progressive deterioration in memory, cognitive functioning, and behaviour and language (World Health Organization, 2017). In 2019, approximately 57 million people worldwide had dementia, with this number expected to surpass 152 million by 2050 (Nichols et al., 2022), largely driven by the world’s ageing population (United Nations, 2022). Consequently, it is recognised as a public health priority (World Health Organization, 2023) and carries substantial economic costs (Wittenberg et al., 2019).

People with dementia are at a disproportionately high risk of mental health problems (Onyike, 2016), with an increased prevalence of anxiety and depression (Leung et al., 2021). Importantly, psychological distress may also be masked by cognitive impairment associated with dementia (Winter et al., 2011), suggesting that such difficulties may go undetected.

Pharmacological interventions are often used to manage dementia symptoms and psychological difficulties, but evidence of their efficacy remains limited (Pai et al., 2024; Rogowska et al., 2022). Non-pharmacological treatments, however, have shown considerable promise. For example, cognitive stimulation therapy, a structured programme of themed activities designed to engage people with dementia in mentally stimulating activities and social interaction (Spector et al., 2003), has been found to improve depression, self-reported quality of life, neuropsychiatric symptoms, global cognition, language, communication and working memory (Desai et al., 2024). While some non-pharmacological approaches can benefit people with dementia, evidence for other non-pharmacological interventions such as talking therapies remains limited.

For the purpose of this review, talking therapies are defined, in line with the National Health Service Talking Therapies service (National Health Service England, 2023), as psychological interventions in which individuals work with a trained professional, either individually or in a group setting, to manage emotional or psychological difficulties. Examples of psychological interventions include, cognitive behavioural therapy (CBT), counselling, mindfulness-based interventions, and other structured approaches aimed at improving mental health outcomes. Although talking therapies demonstrate strong evidence in the general population (Clark, 2018), research on their effectiveness in dementia demonstrates mixed findings. A meta-analysis found that cognitive behavioural therapies have a small positive effect on depression and may improve quality of life, but evidence for anxiety is lacking. Counselling showed little impact on depression, whilst support for mindfulness-based interventions and other therapeutic interventions remains limited (Orgeta et al., 2022). Robinson and Moghaddam (2023) found small positive effects for psychological interventions but highlighted the low quality of existing research. The authors suggested that adapting existing therapies can improve efficacy, for example through slowing the pace of therapy, simplifying language, using repetition, reducing distraction and involving caregivers. Additionally, a recent scoping review reported the effectiveness of CBT-informed interventions in reducing depression symptoms for people with dementia, however, found that anxiety was less frequently measured in studies, with no significant benefit for people with dementia (Charlesworth et al., 2025).

Current literature lacks robust evidence for talking therapies in dementia and highlights the need for adaptation as many people with dementia are missing out on psychological therapy (Bell et al., 2022). According to the UK’s National Institute for Health and Care Excellence guidance (NICE, 2019), interventions for people with dementia should be, “tailored to their preferences,” and therefore be, “feasible, acceptable, enjoyable, suitable and helpful”. Therefore, this study aims to undertake a systematic review and thematic meta-synthesis to explore the experiences of people with dementia in accessing and engaging in talking therapies for mental health difficulties, including studies of both individual and group formats to maximise available insights and incorporating the perspectives of people with dementia, their caregivers and dementia care professionals.

## Materials & Methods

This review was registered on PROSPERO (registration number: CRD42022362418) and follows the Preferred Reporting Items for Systematic Reviews and Meta-Analyses (PRISMA) guidelines (Page et al., 2021).

### Eligibility Criteria

We included:

(1) Empirical, peer-reviewed studies only.
(2) Research which used either an entirely qualitative analytical approach, or which at least included qualitative analysis within a broader mixed methodology analysis.
(3) Work focussed on accessing, delivering or taking part in talking therapies from the perspective of people with dementia, their caregivers or professionals. This included studies containing themes related to talking therapies which had been delivered within the context of broader intervention packages.
(4) Only English language studies (due to a lack of available translation resources).

### Information Sources

We searched PsychINFO, CINAHL and Web of Science in March 2024, along with reference lists of included studies.

### Search Strategy

The search strategy was developed with clinical and dementia research experts. Search terms related to talking therapies were based on therapies offered by the National Health Service in the UK (National Health Service, 2022), those listed in the National Health Service Talking Therapies for anxiety and depression Manual (National Collaborating Centre for Mental Health, 2018) and therapies mentioned in existing literature, as well as generic terms associated with therapy and psychotherapy. No date restrictions were applied. The full search strategy is outlined in Appendix A.

### Study Selection

Search results were combined and duplicates removed using EndNote (Clarivate, 2013) and were also checked manually by the first author. The records were then uploaded to Rayyan (Ouzzani et al., 2016) for screening of abstracts and titles using the eligibility criteria by the first author. 10% of abstracts and titles were also blindly screened by the second author. There was 96% agreement and any discrepancies were resolved through discussion. Full texts of the remaining papers were screened by the author, with the reason for exclusion recorded in each case.

### Quality Assessment

Quality assessment was conducted using a revised version of the Critical Appraisal Skills Programme (CASP) checklist for qualitative research (Critical Appraisal Skills Programme, 2024). An additional item was added to the checklist and was used for each paper to evaluate the theoretical underpinnings of the qualitative studies, “Are the study’s theoretical underpinnings (e.g. ontological and epistemological assumptions; guiding theoretical framework(s)) clear, consistent and conceptually coherent?” (Long et al., 2020).

The quality assessment criteria are outlined in Appendix B.

Where studies deployed mixed methods, only the quality of the qualitative elements of each study was appraised. Although the CASP checklist does not define a scoring system, a score was derived for each paper based on the proportion of items rated as ‘yes.’ As per recommendations (Long et al., 2020), the studies were also classified as high (scores from 8 to 10), medium (scores from 5 to 7) or low quality (scores from 0 to 4). The first author appraised all 15 studies. The second author also appraised two of the studies. Any disagreements were resolved through discussion.

### Synthesis of Results

Synthesis followed the guidance for thematic synthesis outlined by Long et al. (2020). Given the paucity of existing research, an inductive approach was used. All text labelled as either ‘results’, ‘findings’, or ‘discussion’ within the papers was extracted and imported into NVivo (Lumivero, 2024). Included studies were read multiple times to allow data familiarisation. Inductive coding of the data from the four papers which were of high quality was conducted and an initial thematic framework was devised, with medium-quality papers coded next and any expansion on existing themes and any additional themes added. Finally, low-quality papers were coded with no additional themes added, but an expansion on existing themes was incorporated where necessary. Where possible, first-order data (i.e., direct quotations from participants) were coded first. Second-order data (i.e., the words and interpretations of the studies’ authors) were then coded. Where papers had collected data from the same interventions and populations, care was taken to avoid coding data more than once. After this, analytical themes were constructed. These constructs can be thought of as third-order data, i.e., the researchers’ subjective interpretation of the primary authors’ subjective interpretations of the views and experiences of participants.

## Results

### Systematic Search Results

**Figure 1.**
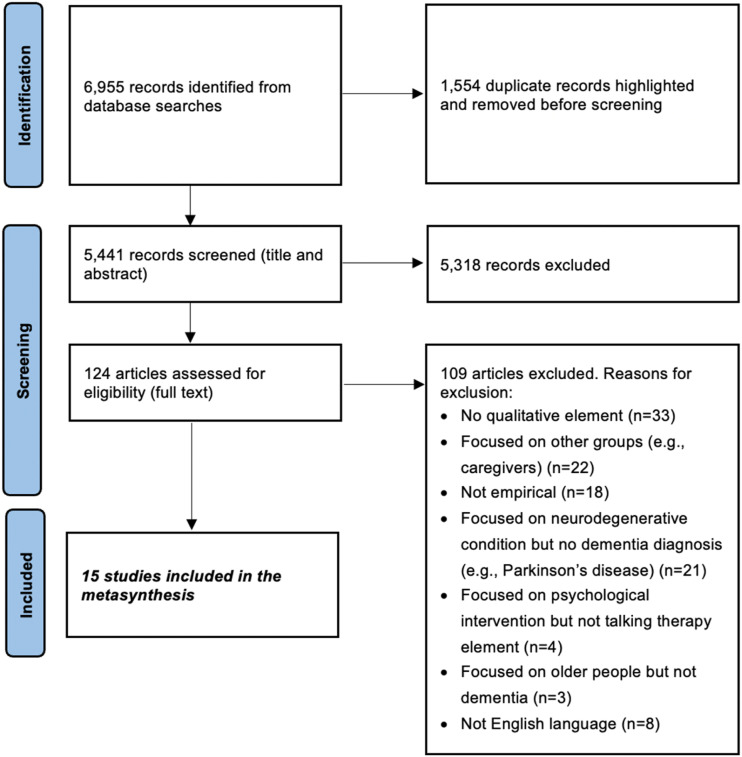
PRISMA flow diagram (Page et al., 2021)

As illustrated in the PRISMA flowchart (see Figure 1), the systematic search generated 6,955 records. After duplicate removal, 5,441 titles and abstracts were screened, 124 full texts were reviewed, and 15 studies were included.

### Study Characteristics

Nine studies were qualitative and six used mixed methods. The majority of studies (*N* = 11) were published between 2018 and 2024. Studies were conducted in the UK (*N* = 10), Denmark (*N* = 2), Sweden (*N* = 2), and the Netherlands (*N* = 1). People with dementia were participants in 13 studies, eight of which also included caregivers/supportive others as participants. Two included both caregivers and professionals, and two others focused solely on the views of professionals. Table 1 provides a detailed overview of the study characteristics.

**Table 1.** Summary of Included Studies

Study ID	First author, year, location	Main focus	Method	Participant group(s)	Participant ethnicity	Dementia type	Clinical dementia diagnoses (Y/N)	Degree of cognitive impairment reported (Y/N)	PLWD age	PLWD gender	PLWD mental health information
1	Baker (2022), UK	CBT therapists’ perspectives on barriers and facilitators to providing therapy to PLWD	Qualitative: Semi-structured interviews. Thematic analysis	IAPT professionals: PWPs (*n* = 7), CBT therapists (*n* = 5), clinical psychologists (*n* = 2)	White British (*n* = 7), Black British (*n* = 2), Asian British (*n* = 2), other ethnic origin (*n* = 3)	Dementia and MCI	NA	N	NA	NA	Not reported
2	Berk, Warmenhoven (2019), NL	PLWD and their partner’s views of a mindfulness-based intervention	Mixed methods: Semi-structured interviews. Deductive content analysis	PLWD (*n* = 7), Partner caregivers (*n* = 7)	Not reported	AD (*n* = 4), VaD (*n* = 2), FTD (*n* = 1)	Y	N	Mean = 71.46	2 female, 5 male	Not reported
3	Birtwell (2018), UK	PLWD’s views of various psychological supports, including talking therapies	Qualitative: Semi-structured interviews. Thematic analysis	PLWD (*n* = 5)	White British (*n* = 3), African Caribbean (*n* = 2)	AD and VaD	Y	N	Range = 58–79	4 female, 1 male	Not reported
4	Blomberg (2024), SE	Views of key stakholders, inc PLWD, on low-intensity behavioral activation intervention	Qualitative: Semi-structured interviews and focus group discussions. Content analysis	Healthcare professionals (*n* = 18), community stakeholders (*n* = 7), people with dementia (*n* = 8), informal caregivers (*n* = 19)	Not reported	AD	Y	N	Mean = 73	4 female, 4 male	Not reported
5	Burns (2005), UK	PLWD’s experiences of psychodynamic interpersonal therapy, including carers’ perspectives on the PLWD’s experiences	Mixed methods: Survey including open-ended items. Comments reported verbatim	PLWD (*n* = 20), Partner caregivers (*n* = 20)	Not reported	‘AD with mild dementia,’ with a ‘clinical dementia rating of 1'	Y	Y	Range = 62–88, Mean = 73.9	10 female, 10 male	Not reported
6	Cheston (2004), UK	Professionals’ perspectives on narrative and emotional change brought about by psychotherapy groups for PLWD	Qualitative: Data comprised notes written after therapeutic sessions. Assimilation analysis	PLWD (*n* = 1)	Not reported	VaD (*n* = 1)	Y	N	‘Late 70s’	1 male	Not reported
7	Craig (2018), UK	PLWD and supportive others’ perspectives on a compassion focused therapy intervention for PLWD	Mixed methods: Semi-structured interviews. Thematic analysis	PLWD (*n* = 5), Supportive others (*n* = 4)	PLWD: White British (*n* = 4), White European (*n* = 1), Other participants’ ethnicities not reported	AD (*n* = 3), VaD (*n* = 1), Mixed AD & VaD (*n* = 1)	Y	Y	Range = 53–83, Mean = 73	4 female, 1 male	Participants must have been above threshold for depression on the CSDD and/or for anxiety on the RAID
8	Douglas (2022), UK	Perspectives of PLWD, carers and professionals’ of a mindfulness-based cognitive therapy intervention for depression in PLWD	Qualitative: Semi-structured interviews. Thematic analysis	PLWD (*n* = 8), Caregivers (*n* = 6), Facilitators (*n* = 4)	PLWD: White British (*n* = 7), Black Caribbean (*n* = 1), Other participants’ ethnicities not reported	AD (*n* = 5), VaD (*n* = 1), Mixed dementia (*n* = 1), unspecified (*n* = 1)	Y	Y	Range = 62–93, Mean = 77.3	7 female, 1 male	Participants must have met criteria for mild depression, based on clinical cut-off score of 9 on the PHQ-9
9	Griffiths (2021), UK	Perspectives of PLWD and their carers on a relational counselling intervention	Qualitative: Semi-structured interviews. Framework analysis	PLWD (*n* = 6), Caregivers (*n* = 23)	PLWD: White British (*n* = 4), White European (*n* = 2), Other participants’ ethnicities not reported	AD (*n* = 4), VaD (*n* = 1), Mixed dementia (*n* = 1)	Y	Y	Range = 61–95, Mean = 81	3 female, 3 male	Not reported
10	Robinson (2023), UK	Analysis of therapeutic change within three PLWD participating in an ACT intervention based on perspectives of ‘psychotherapy experts’	Mixed methods: hermeneutic single-case efficacy design series based on quantitative and qualitative data including semistructured change interviews	PLWD (*n* = 3)	PLWD: White British (*n* = 3), Carers: White British (*n* = 3)	AD (*n* = 1), Mixed AD and VaD (*n* = 1), VaD (*n* = 1)	Y	N	Range = 71–90, Mean = 82	3 female	Participants must have met criteria for ‘clinically significant level of psychological distress,’ based on scores on PHQ-9 (≥10) and GAD-7 (≥8)
11	Sass (2022), UK	Perspectives of PLWD, their carers and professionals on a relational counselling intervention	Qualitative: Semi-structured interviews. Framework analysis	PLWD (*n* = 6), Caregivers (*n* = 23), Counsellor (*n* = 1), Service co-ordinator (*n* = 1)	PLWD: White British (*n* = 4), White European (*n* = 2), Other participants’ ethnicities not reported	AD (*n* = 4), VaD (*n* = 1), Mixed dementia (*n* = 1)	Y	N	Range = 61–95, Mean = 81	3 female, 1 male	Not reported
12	Skov (2022), DK	Views of PLWD and their carers on a multicomponent psychosocial intervention for people with early-stage dementia including counselling	Mixed methods: Interviews (structure not reported) Thematic analysis	PLWD (*n* = 14), Caregivers (*n* = 13)	Not reported	Not specified for qualitative element; overall participants: AD (*n* = 23), VaD (*n* = 4), Mixed AD and VaD (*n* = 1), Lewy body dementia (*n* = 3), Other (e.g., Parkinson’s with dementia diagnosis) (*n* = 4), unknown (*n* = 1)	Y	Y	Range = 60–86, Mean = 74.4	1 female, 13 male	Not reported
13	Sørensen (2008), DK	PLWD and their carers’ views on a counselling intervention for individuals with mild Alzheimer’s disease and their carers	Qualitative: Semi-structured interviews. Specific methodology unclear, though grounded theory mentioned	PLWD (*n* = 10), Partners (*n* = 10)	Not reported	Participants described as having ‘Mild AD’	Y	Y	Range = 65–81, Mean = 73.4	5 female, 5 male	Not reported
14	Svedin (2023), SE	Healthcare professionals and community stakeholders’ perspectives on barriers and facilitators to delivering a behavioural activation intervention	Qualitative: Semi-structured interviews and focus group discussions. Content analysis	Healthcare professionals (*n* = 18), community stakeholders (*n* = 7)	Not reported	‘Mild to moderate dementia’	NA	N	NA	NA	Depression
15	Watkins (2006), UK	Professionals’ perspectives on changes in awareness brought about by psychotherapy groups for PLWD	Mixed methods: Tapes and transcripts of therapeutic sessions. Assimilation analysis	PLWD (*n* = 1)	Not reported	AD (*n* = 1)	Y	N	76	1 male	Depression and anxiety measured using the CSDD and the RAID

*Note.* Abbreviations: Acceptance and Commitment Therapy (ACT), Alzheimer’s disease (AD), Critical Appraisal Skills Programme (CASP), Cognitive behavioural therapy (CBT), Compassion-focused therapy (CFT), Cornell Scale for Depression in Dementia (CSDD), Denmark (DK), Frontotemporal dementia (FTD), Generalised Anxiety Disorder Questionnaire (GAD), Improving Access to Psychological Therapies (IAPT), Mild cognitive impairment (MCI), Netherlands (NL), Not applicable (NA), Patient Health Questionnaire (PHQ), People living with dementia (PLWD), Psychological Wellbeing Practitioner (PWP), Rating Anxiety in Dementia (RAID), Sweden (SE), Vascular dementia (VaD).

Two UK studies analysed the same intervention; Sass et al. (2022) examined professionals alongside people with dementia and family caregivers, and Griffiths et al. (2021) included the views of people with dementia and family caregivers only. Given that the demographics of the people with dementia and family caregivers were the same across the two studies, it was judged as highly likely that there was overlap in the people with dementia and caregivers who took part in the two studies. Similarly, two Swedish studies also examined the same intervention; Blomberg et al. (2024) examined the views of healthcare professionals, community stakeholders, people with dementia, and informal caregivers as participants, whilst Svedin et al. (2023) focused on the perspectives of healthcare professionals and community stakeholders only. Community stakeholders were defined as individuals involved in non-profit dementia and caregiver organisations (Blomberg et al., 2024). Given that the same numbers of healthcare professionals and community stakeholder groups were referred to in these studies, it was also judged as highly likely that they overlapped. Taking these potential duplications into account, it was calculated that 88 people with dementia, 96 caregivers, family members or supportive others, 38 professionals and seven community stakeholders took part in the qualitative components of the studies. Where it was unclear if participants within mixed methods studies took part in both the quantitative and qualitative elements of the paper, demographic information is reported for the study as a whole. Additionally, five of the studies described using a participatory approach to some extent (Birtwell & Dubrow-Marshall, 2018; Blomberg et al., 2024; Craig et al., 2018; Douglas et al., 2022; Svedin et al., 2023).

Across the 13 studies which looked at the experiences of people with dementia, 43 females and 45 males took part. The mean age of the people with dementia ranged from 73 to 82. Six studies reported on ethnicity, with 21 participants described as White British, three described as White European, two as African Caribbean and one as Black Caribbean.

Interventions included, acceptance and commitment therapy (ACT; *N* = 2), mindfulness-based approaches (*N* = 2), cognitive behavioural therapy (CBT; *N* = 1), behavioural activation (*N* = 1), compassion-focused therapy (*N* = 1), psychodynamic interpersonal therapy (*N* = 1), group psychotherapy (*N* = 2), multi-component intervention with a counselling element (*N* = 1), and multiple interventions (*N* = 1).

All studies which incorporated people with dementia as participants (*N* = 13) specified that these individuals must have a dementia diagnosis. However, only three of the studies specified that participants must have a particular mental health diagnosis or meet a specific threshold of symptoms in order to take part. See Table 1 for additional details.

### Quality Assessment

Overall, five studies were deemed to be of high quality, with nine papers deemed to be of medium quality, and one of low quality. No studies were deemed to have met all of the criteria. Table 2 provides an overview of the quality assessment ratings.

**Table 2.** Quality Assessment Ratings (Critical Appraisal Skills Programme, 2024; Long et al., 2020)

Study ID	Authors	Year	Q1	Q2	Q3	Q4	Q5	Q6	Q7	Q8	Q9	Additional Item	Overall quality score	Rating descriptor
1	Baker, Brede, Cooper, Charlesworth, & Stott	2022	Y	Y	C	Y	Y	Y	Y	Y	Y	C	8	High
2	Berk, Warmenhoven, Stiekema, Van Oorsouw, Van Os, de Vugt, & Van Boxtel	2019	Y	Y	C	Y	Y	C	Y	Y	Y	C	7	Medium
3	Birtwell & Dubrow-Marshall	2018	Y	Y	C	Y	Y	Y	Y	Y	Y	C	8	High
4	Blomberg, O., Svedin, F., Farrand, P., Brantnell, A., von Essen, L., Patriksson Karlsson, J., … & Woodford, J	2024	Y	Y	C	Y	Y	C	Y	Y	Y	C	7	Medium
5	Burns, Guthrie, Marino-Francis, Busby, Morris, Russell, … & Byrne	2005	Y	Y	C	Y	C	C	C	C	Y	C	4	Low
6	Cheston, Jones, Gilliard, & Moriarty	2004	Y	Y	Y	Y	C	C	C	Y	Y	Y	7	Medium
7	Craig, Hiskey, Royan, Poz, & Spector	2018	Y	Y	C	Y	C	C	Y	C	Y	C	5	Medium
8	Douglas, Stott, Spector, Brede, Hanratty, Charlesworth, … & Aguirre	2022	Y	Y	Y	Y	Y	Y	Y	C	Y	Y	9	High
9	Griffiths, Shoesmith, Sass, Nicholson, & Charura	2021	Y	Y	C	Y	C	C	Y	C	Y	C	5	Medium
10	Robinson, A., De Boos, D., & Moghaddam, N	2023	Y	Y	Y	Y	Y	Y	Y	Y	Y	C	9	High
11	Sass, Griffiths, Shoesmith, Charura, & Nicholson	2022	Y	Y	C	Y	C	C	Y	C	Y	C	5	Medium
12	Skov, Nielsen, Krølner, Øksnebjerg, & Rønbøl Lauridsen	2022	Y	Y	C	Y	C	Y	Y	Y	Y	C	7	Medium
13	Sørensen, Waldorff, & Waldemar	2008	Y	Y	C	Y	C	Y	Y	C	Y	C	6	Medium
14	Svedin, F., Blomberg, O., Brantnell, A., Farrand, P., Åberg, A. C., & Woodford, J	2023	Y	Y	C	Y	Y	C	Y	Y	Y	C	8	Medium
15	Watkins, Cheston, Jones, & Gilliard	2006	Y	Y	Y	Y	Y	C	C	Y	Y	Y	8	High

*Note.* Values for Question 10 of the CASP Tool (‘How valuable is the research?’) are not included in the table above as this item asks for an opinion rather than a yes/no value. The CASP tool does not designate specific ranges for low, medium and high quality studies, therefore the following ranges were used: 0 to 4 (low), 5 to 7 (medium) and 8 to 10 (high). Y = yes, C = cannot tell.

### Thematic Synthesis

**Figure 2.**
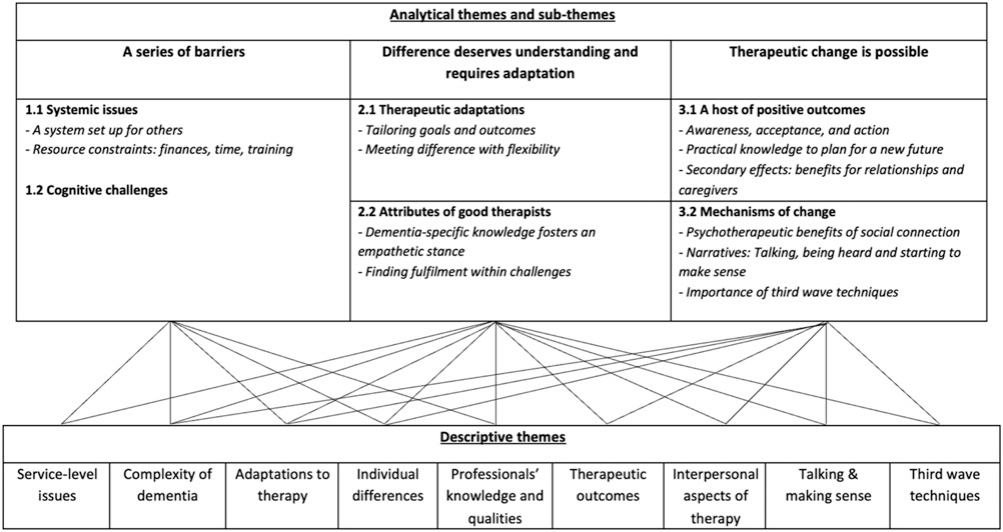
Thematic map of findings: Descriptive themes and analytical themes and sub-themes

Three superordinate themes “A series of barriers”, “Difference deserves understanding and requires adaptation” and “Therapeutic change is possible” and associated sub themes were derived. Figure 2 outlines a thematic map. Direct participant quotes are italicised with quotation marks and author interpretations as non-italicised with quotation marks. Unique study IDs (see Table 1) are included after quotations to indicate the original paper from which these were derived.

## A Series of Barriers

This theme highlights the challenges people with dementia experience in accessing and taking part in talking therapies, focussing on both systemic and cognitive barriers.

### Systemic Issues

Several papers described systems and processes not designed for people with dementia. In some cases, referrals were not made at all, potentially due to dementia masking mental health difficulties (*“The GP [primary care physician] might not pick up that actually they are depressed”*^1^), or the perception that taking part in talking therapies would not be possible for people with dementia, with staff within UK primary care therapy services noting a lack of referrals possibly due to societal stigma, and negative assumptions from referrers, often primary care physicians:

“There was just like this underlying type of ‘well it’s not going to be successful anyway because people need these skills to be able to engage in therapy and they don’t have those skills’”^1^.

In others, it was suggested that referrals may not be ‘suitable,’ with professionals stating that careful consideration should be given to, *“how and who [we are] letting into the service,”*^11^ or that referrals may be lacking in sufficient detail: “it would have been helpful if more information about how aware participants were of their diagnosis had been included in participant referrals” ^8^.

Stigma was also highlighted as a barrier to accessing services in Sweden, with, “both dementia and mental health difficulties … considered to be stigmatized in society”^14^.

Participants suggested that rigid criteria for assessing therapy effectiveness may be a barrier: *“We’d be seeing patients that we can’t count towards recovery … this would reduce our recovery rates, which would therefore forward impact how effective our service looks to commissioners”*^1^. The need for flexibility in evaluating the “therapeutic benefit”^11^ of interventions due to dementia progression impacting the “ability to engage”^1^, and developing new outcome measures to understand which members of the “dyad”^2^ actually benefit from such interventions was highlighted.

There was a sense of resource scarcity, with a Swedish study noting that services were “limited or non-existent in smaller cities”^14^. People with dementia also noticed the effects of “financial cuts and eligibility for support”^3^. Participants in a study based in a UK NHS service, voiced a “wish for more support”^7^, whilst in a Netherlands-based study, participants argued that “health insurance should cover [mindfulness] training for caregivers and PWD [people with dementia]”^2^. Similarly, professionals suggested that interventions for this population may require them to “change [their] ways of working”^14^ such as offering a, “greater number of sessions”^1^ than was possible due to *“limited time and limited resources”*^1^, and that interventions felt hampered by *“time constraints”*^8^.

Several papers also highlighted a lack of dementia-specific training for staff, with one noting that “lack of resources and training resulted in staff feeling unsupported”^1^ and another suggesting that “lack of knowledge or education in dementia … may lead to lack of confidence and trust”^14^.

Outside the UK, it was suggested that a lack of well-translated materials also hindered therapy, due to resources being inadequately “*translated from English”*^4^.

### Cognitive Challenges

People with dementia and their caregivers identified cognitive challenges with therapy engagement. Several studies described individuals lacking insight into their cognitive difficulties. Some participants found therapy *“too difficult”*^3^. The complexity of the materials in both CBT *(“I – I don’t think I would be able to do that”*^3^*)* and mindfulness (“due to the literacy skills”^8^) was also noted. Difficulties with concentration were also described: *“I did my best to try and I listened to the CD…I tried to concentrate, it’s something that I wanted to be able to help, [but]…worries or whatever, start coming in and mixing it up”*^8^. Difficulties in motivation and executive function, such as a “lack of ability to take initiative”^14^ were also highlighted by professionals.

Memory issues were also noted, with participants struggling to retain information about the interventions themselves or their providers. Professionals suggested that memory issues were “potentially influencing the validity of measures”^1^. Moreover, it was suggested that memory difficulties may affect efficacy and ability to practice skills. Participants found interventions beneficial, but had trouble recounting specific elements or techniques: “*There was something, but guess what? I can’t remember what it was!”*^*7*^; *“…when you’ve got someone with memory problems like she’s got, by the time she came home from the class she’d forgotten really, what they did there”*^8^. Several studies suggested that talking therapies may be more relevant for individuals in the *“early”*^4^ stages of dementia, as those whose condition had “progressed”^10^ may struggle.

Positively, several studies identified key intervention elements that mitigated these challenges. For example, behavioural activation’s highly structured and “concrete”^14^ approach was described as an accessible tool that was “well received”^4^, successfully bypassing the abstract demands of some other therapies. Second, the embodied nature of mindfulness-based techniques appeared beneficial, potentially due to reduced reliance upon complex language or memory recall, making these easier to grasp. Deep-breathing, for example, was cited as something which actually helped people with dementia “feel less distracted and … calmer”^8^.

## Difference Deserves Understanding and Requires Adaptation

Across studies, participants discussed a need for understanding and adaptation to prevent excluding people with dementia from therapy. This involved both therapeutic adaptations and essential therapist attributes.

### Therapeutic Adaptations

A flexible therapeutic stance was widely recommended, particularly when using rigid models and protocols. Due to concerns about outcome measures affecting service uptake, several papers proposed adapting therapeutic goals. One counsellor suggested that focusing on reducing fears and improving understanding of their current situations may be more appropriate goals: *“my aim is to make things better for them…to take away, some of the scaredness somehow to sort of understand what they’re going through”*^11^. The authors of another paper cautioned against using common mental health constructs like “*distress”*^8^.

A mixed methods study found “No improvement … on the majority of [standardised] outcome measures”^5^ but reported therapists as a “major source of support”^5^ for both caregivers and people with dementia. Similarly, standardised self-report measures may be inadequate due to the complexity of the language used “in the context of memory impairment”^10^.

Two studies^6,15^ suggested that the “assimilation of problematic experiences”^6^ or “coming to terms” with dementia may be a more appropriate central concern when working with people with dementia than the symptom-focused approach traditionally used in talking therapies. Specifically, they argue that the “adequate processing of … painful, or problematic, experiences,” associated with having a diagnosis of dementia with the aim of incorporating these into one’s, “existing beliefs”^6^ is a particularly important objective for talking therapies with these populations. They found that they were able to measure this process using the Assimilation of Problematic Experiences Scale (APES).

Flexibility was also seen as important for effective therapy. Therapists suggested that “longer”^8^ sessions to accommodate cognitive challenges, and the use of “follow-up sessions…to ‘embed’ practice”^8^. Professionals also discussed the benefit of meeting with participants in advance of therapy in order to set expectations around what therapy would involve, give prospective clients the chance to familiarise themselves with the environment, and to “address any initial concerns”^11^. Consistency and routine were highlighted by therapists as beneficial to get participants “in the zone”^8^ and by people with dementia who “expressed preferences for structured guidance at the same time on a weekly basis”^4^.

Where possible, adaptation of therapeutic materials was found to increase the acceptability of interventions for people with dementia and their caregivers, as well as improving accessibility and enhancing the engagement of people with dementia in talking therapies. This included focusing on, “shorter, more immediate and less abstract exercises”^10^, whilst making materials more visual, incorporating videos and recordings of techniques, building exercises into everyday tasks, and using memory aids between sessions. One study highlighted feedback from participants who suggested that materials depicted “stereotypically older adults”^4^ and that content felt “old fashioned”^4^, with one participant saying facilitators were, *“speaking to you like a child”*^4^. It was suggested that this may, “negatively [impact] intervention relevancy and potentially [exacerbate] stigma surrounding dementia and older age”^4^.

Supportive others (e.g., family members and caregivers) played an important role in facilitating the therapeutic process by encouraging attendance, helping to maintain continuity and routine, and assisting with tasks outside of the therapeutic space itself. Some people with dementia wanted caregivers involved “in order to have access to the same knowledge”^13^ or to be the *“instigator”*^2^, reminding them to engage in home practice and facilitation “conversations about therapy and [support] with between-session tasks”^10^. Caregivers also helped assessments by providing insights into individuals’ wider contexts and “*how it is impacting the family system”*^1^. However, Svedin et al. (2023) highlight a risk related to involving caregivers, who may experience feelings of “failure”^14^ if the intervention is perceived as unsuccessful.

Finally, professionals in several studies highlighted the need to adapt environments, considering, “sensory and physical difficulties”^8^ and ensuring “accessible buildings”^1^. One study highlighted that “enabling dyads to attend the centre together and access counselling [together]”^11^ was perceived as a supportive therapeutic environment.

### Attributes of Good Therapists

Several studies (*n* = 4) suggested therapists’ dementia-specific knowledge was critical to “tailor”^1^ their practice to avoid individuals feeling “unsupported”^1^. Participants and their caregivers suggested that having a “knowledgeable listener”^9^ brought something more than a *“general”*^*9*^ approach. This “knowledge and experience”^4^ was seen as key to “build a trusting care relationship”^4^. The ability to build such relationships with not only people with dementia, but with the “dyad”^14^ was critical.

Therapists suggested that “Being able to separate behaviours associated with dementia and the person themselves was beneficial”^11^. Others suggested that sadness might be more prevalent in this population due to dementia-related losses, and that professionals should help clients be “guided in this process”^2^. It was highlighted that such knowledge should be based on experience working with clients and not just formal training in order to develop a *“capacity to appreciate the lived experience of their clients”*^11^ and to, *“work flexibly to the needs and abilities of people affected by dementia”*^11^. Although some professionals argued that, “cognitive impairment had little impact”^1^ on therapy, there was a general agreement that professionals should understand that dementia presentations are highly heterogeneous and that people will have varied functioning in different stages which impact therapy. For example, working on practical planning to instil a “sense of control”^15^ may be helpful for individuals who have, “developed an understanding of their memory difficulties”^15^ but a more, “exploratory”^15^ approach may be required for individuals at earlier stages of awareness and acceptance, or who have reduced insight. Overall, knowledgeable therapists were described as creating a *“safe”*^14^, *“harmonious and friendly”*^2^ therapeutic space.

Multiple studies acknowledged that working with people with dementia can be *“challenging”*^1^ but that it was ultimately *“rewarding”*^1^ for therapists who felt an *“ethical responsibility”*^8^ to provide adapted talking therapy. There was also a sense that therapists working with this population should understand that there may be a greater “emotional impact”^11^ upon them in comparison to working with the general population. This might be driven by the “stress”^1^ of adapting interventions, by the fact that participants may have trouble recognising staff and that forming relationships may be difficult, or because achieving psychotherapeutic change may not be possible in the same way as it might for the general population.

## Therapeutic Change is Possible

Despite several barriers and challenges, the message from many professionals, caregivers and people with dementia was that talking therapies can be helpful and possible as long as adaptations are made.

### A Host of Positive Outcomes

A key outcome described across several studies was that participants developed a greater awareness of their situation, potentially moving people with dementia “towards acceptance and asserting control”^3^ and an ability to “make peace with their circumstances”^9^, leading to engagement in valued activities. This was consistent across papers exploring mindfulness-based interventions, behavioural activation, counselling, compassion-focused therapy and group psychotherapy.

Participants often “began to articulate an awareness of [their] diagnosis”^15^ and became “more open to their difficulties”^13^ during therapy. Even when participants could not consciously articulate the change, an increase in awareness occurred: “Despite their forgetfulness about the intervention, they both talked about their memory problems as being a consequence of the disease in the second interview after the intervention”^13^. This awareness often moved towards acceptance: *“I have accepted the fact that I have a ‘memory problem’ and I am happy being me”*^7^. Such acceptance appeared to help individuals and their caregivers reduce *“self-blame”*^7^, increase *“self-compassion”*^*8*^, improve “mood and coping”^9^ and, “resilience”^2^. It allowed participants to gain a sense of control, as well as developing a more optimistic view of the future: *“I’m looking forward to sort of, the future now”*^9^. Related to this, several studies, including those examining behavioural activation, counselling and mindfulness-based approaches, described how this, in turn, allowed participants to “recognise and live in line”^10^ with existing goals as well as to *“set new goals”*^14^. This helped people with dementia to engage in values-driven activities and gain a sense of achievement: *“The therapy has made my mum realise that she’s still got things she wants to do in life”*^10^.

In addition to awareness and acceptance, participants in several studies benefitted from psychoeducational components, learning about dementia, the “limitations associated with their cognitive impairment”^9^ and, “anticipated decline”^9^. Participants and their caregivers also gained specific information around the “legal aspects”^13^ associated with the condition, allowing them to better plan for the future, for example working on a “*power of attorney”*^13^.

Whilst people with dementia benefited directly, those therapies that had a relational focus or involved both people with dementia and their caregivers, often led to relationship improvements. A professional shared: *“Once I met a caregiver and I just listened to her, I did not say anything, because I did not know much then. But then she came back and she said ‘Thank you, I remember you. Everything was such chaos, but you were there and listened to me’”*^14^. People with dementia often gained awareness of their roles in relationships, adopting “new role[s]”^9^ within relationships which had changed in light of dementia diagnoses, with one participant mentioning they had started washing up on an evening: “*so when tea’s finished she can get ready watching the television”*^9^. In some relationships, interventions improved and “[facilitated] conversations”^14^ and gave couples the skills to communicate more clearly, for example, one person mentioned that their partner used to talk very fast and now “*She says, ‘Let me explain,’ and then she does explain it. And she really does. And that makes her so much happier, and me too. So that works”*^2^. In others, it led to, “a willingness to explore love and connection in the changed relationship”^2^ as well as discovering “mutual communication about the consequences of the disease”^13^. Other times, taking part together provided *“quality time”*^7^ for dyads, and seeing their loved one’s own psychological health improve had a positive effect on caregivers’ own wellbeing: *“It’s [the course] made me a little bit less stressed about her...I think, for me, it’s helped because she is less depressed, and she’s lighter, so it, kind of, takes something off my shoulders”*^8^.

### Mechanisms of Change

Across the studies, it was suggested that mechanisms of change for people with dementia may differ from the general population.

The social aspects of therapy were particularly impactful, especially in group settings. For some people with dementia, group participation provided a way to, “assess … and compare”^3^ their impairment with other people with dementia. This provided “some sense of control and reassurance in their ability to cope”^3^. Group therapy also provided “a sense of belonging”^8^ and not “feeling stupid”^8^ when talking to peers in comparison to an *“outside world”*^8^. For some, it facilitated learning from others, for example, learning *“what will happen”*^2^ in the future. This helped them address *“the fear”*^*2*^ and to learn from their peers by *“hear[ing] from other people how they felt and what they experienced”*^2^. Addressing these “common issues of concern”^6^ also allowed individuals to “[foster] a sense of shared identity”^6^. Feedback from one study found that individuals with dementia viewed talking therapies as something that “influenced self-concept and social identity”^3^. For others, human connection alleviated the loneliness with some *“looking forward”*^8^ to sessions because they lived alone. Even interactions with wider service members created a sense of community: “*you got to know everybody do you know what I mean?”*^11^.

Talking therapy also helped many individuals make sense of their emotions and situations. It addressed the “scant opportunities they had for sharing and relating to others”^11^ allowing them to *“let things out that you have inside”*^3^. For some, therapy provided a space to be listened to: “*somebody who is actually going to listen to what I say, and take some notice”*^9^. Therapy also provided an outlet for things they might otherwise hold back. These included, for example, not having previous experience of discussing emotional topics (*“I’m not used to people asking sort of personal questions … but I got used to it as time went on”*^10^), the belief that people would not want to listen (*“other people don’t want to hear me talking about it all the time”*^11^), that their situation was not *“worth talking about”*^13^ or the perception that it is selfish to talk about oneself *(“I realised … wait a minute I’m not being selfish, I need this time for me, and basically doing this therapy gave me permission to say … uhm … sorry I can’t do it”*^7^*)*. Although such conversations were described as “painful”^5^ or leading to “sadness”^2^, there was a strong sense across the studies that experiencing such emotions was an “intra-psychic cost”^15^, which was a necessary step towards processing dementia’s effects and supporting wellbeing.

Several studies also mentioned the importance of figurative language when talking. Analogy and metaphor helped convey therapeutic techniques, and helped individuals to process their situations through “indirect expression”^6^.

In addition to fostering acceptance to values-driven activities, third wave techniques like grounding and mindfulness proved beneficial. Breathing exercises were particularly helpful, with one participant stating: *“And then I just focus on my breath. And I know I can’t do anything to change this. This doesn’t make it painless, but it helps me to keep going”*^2^. Mindfulness’s embodied nature made it accessible, as it didn’t rely on complex language or materials in a way that other talking therapies might. Techniques like “mindfully doing the dishes”^8^ were easy to incorporate into daily life.

Mindfulness techniques also helped participants focus on the present moment, in stark contrast to ruminating on loss or worrying about the future, phenomena which appeared common amongst people with dementia. Attending to the present moment was described as “*soothing”*^8^ leading to “increased awareness and spending less time on automatic pilot”^2^, possibly aiding people with dementia in “concentrating more on their actions”^8^. Counsellors, also described the “value [of] transforming the client’s perspective in that moment”^9^ and, “thinking less about the future implications”^11^. This suggests that a mindfulness-based approach may be incorporated into a range of therapies.

Additionally, metacognitive awareness was another benefit with participants: “noticing thoughts without getting caught up in them”^14^, gaining the ability to “defuse”^10^ from thoughts and being able to, *“let [them] go”*^2^. These techniques helped participants develop “self-kindness”^8^ and confidence: *“[the sessions] stopped me thinking that I was absolutely useless… It made me feel better in myself”*^8^.

Whilst only one study focussed on compassion-focused therapy, the results were promising, helping to “regulate threat-based feelings, and [increase] the activation of the soothing system”^7^, whilst also improving self-awareness and acceptance.

## Discussion

We synthesised 15 qualitative studies on the experiences of people with dementia in accessing and taking part in talking therapies, incorporating the views of people with dementia, their family members and caregivers, as well as relevant professionals, producing a combined understanding of this population’s experiences.

A key finding was that accessing talking therapies was inherently difficult. Current systems are not designed for individuals with dementia. Referrals are often infrequent, with professionals often assuming that services are unable to meet dementia-related needs or that psychological distress should simply be accepted (Burns, 2017; Collins & Corna, 2018). Resource constraints and a lack of dementia-specific training among professionals also contributed to this issue, in line with previous research (Smith et al., 2019). Rigid use of standard outcome measures often not designed for people with dementia, can make interventions appear less effective. This matters for commissioners, who fund services based on these measures; for therapists, who rely on them to guide care; and for people with dementia, who may have limited access or whose progress may be undervalued. Tailored measures are needed to capture meaningful benefits. Collectively, these findings are supported by research exploring therapists’ perceptions of providing CBT interventions for people with dementia or mild cognitive impairment, which identified similar barriers including stigma towards dementia and mental health, staff pressure without adequate support, exclusion based on diagnosis, and inflexibility within services (Baker et al., 2022).

However, results highlighted that talking therapies can benefit people with dementia in several ways, including improving relationships with others, developing greater awareness of their circumstances, providing an emotional outlet, enhancing self-awareness and fostering acceptance, leading to engagement in valued activities. Positive changes were reported, even when participants struggled with recall and articulation, with therapy helping individuals confront and accept situations and also benefiting caregivers. Group therapy allowed individuals to learn from each other and form shared identities. With loneliness acutely prevalent amongst older people (Chawla et al., 2021), and social connection shown to be associated with slower cognitive decline (Samtani et al., 2022), this may be particularly important. The act of talking was also cited as specifically important for this population, allowing individuals to feel genuinely listened to, facilitating the processing of painful subject matter, and allowing individuals to begin making sense of overwhelming situations. These findings align with previous literature reporting improvements in depressive and anxiety symptoms among individuals with early to moderate dementia, as well as enhanced mood and responsiveness in those with more advanced dementia stages who accessed therapeutic counselling (Mathews et al., 2024). It is important to note that the present study adds value to the existing literature by providing evidence that talking therapies can foster self-awareness, acceptance, and emotional expression among people with dementia, which are benefits that extend beyond symptom reduction typically reported in previous research.

Third-wave techniques offered several benefits for people with dementia, including greater acceptance, emotional regulation, and reduced rumination. They also promoted self-awareness, focus on the presence, self-kindness, confidence and emotional soothing. Although evidence for the general efficacy of mindfulness for people with dementia still remains limited (Nagaoka et al., 2021) and requires further exploration, several mechanisms of change were identified in the literature explored here in relation to overarching policy, outcome measurement and service provision, as well as therapy delivery.

Several therapeutic adaptations were identified to maximise access to talking therapies and its benefits. These included changes in session length, addition of follow-up contacts and reminders between sessions, the use of repetition and clearer communication, as well as the incorporation of adapted materials. This aligns with previous research that suggests the efficacy of such modifications (Robinson & Moghaddam, 2023).

Importantly, professionals also suggested moving away from standardised measures, shifting focus from constructs such as anxiety and depression to the more general concept of distress, and measuring success through the degree to which individuals assimilate or come to terms with life changes (Stiles, 2001). This finding extends existing research by suggesting a shift from practical or procedural adaptations, such as altering session length, incorporating follow-ups, and providing reminders, toward a more conceptual adaptation that rethinks the goals of therapy. Rather than evaluating outcomes solely through symptom reduction, practitioners emphasise looking at personal meaning, adjustment, and lived experience as indicators of progress. This perspective highlights how service design can evolve not only to practically accommodate different needs but also to reshape how therapeutic success is understood in more inclusive and person-centred ways.

Studies suggested that it would be misleading to suggest that all people with dementia can take part in such interventions without difficulty. Memory and processing challenges were significant and individual differences, including individuals’ type and stage of dementia, as well as variation in cognitive reserve (Stern, 2009), will influence this. However, this review provides tentative evidence for the acceptability and potential benefit of talking therapies, in line with recent evidence showing significant reductions in depression and anxiety symptomatology for this population (Bell et al., 2022).

### Clinical Implications

In line with quantitative studies providing tentative evidence for talking therapies in people with dementia (Bell et al., 2022; Orgeta et al., 2022; Robinson & Moghaddam, 2023), the present findings suggest talking therapies may offer meaningful benefits within this population. The findings of this review highlight practical considerations for improving access, engagement, and outcomes for people with dementia experiencing mental health difficulties. Key implications include:

#### Therapeutic Engagement

Clinicians should challenge assumptions that people with dementia cannot engage in therapeutic activities. The evidence here indicates that, with appropriate support and adapted approaches, meaningful engagement is possible. Services should prioritise relationship-building, allow flexible pacing, and ensure continuity, so that therapeutic potential is not underestimated.

#### Outcome Measurement

Standardised outcome measures may fail to capture meaningful progress for people with dementia, risking underestimation of therapeutic benefit. Whilst useful, outcome measures should not be used to the detriment of approaching unique therapeutic cases in an individualised manner (Evans, 2012). Clinicians should use or develop bespoke measures and consider broader wellbeing constructs beyond symptoms of depression and anxiety.

#### Service Flexibility and Support

Commissioners and services should ensure that services are funded and staffed adequately, and that clinicians receive dementia-specific training and supervision, so therapies can be effectively adapted and delivered for people with dementia.

#### Individualised Care

Those delivering interventions should recognise the diversity of cognitive abilities in people with dementia. Services should involve people with dementia and their caregivers in research and service development to improve accessibility, relevance, and outcomes.

### Strengths and Limitations

To our knowledge, this is the first review of its kind, offering insights into the lived experiences of people with dementia, as well as of the individuals who support and deliver interventions with them. Notably, 11 of the included studies were published from 2018 onwards, with seven published from 2022 onwards. Whilst the small number of papers demonstrates a research gap, this review provides an insight into a growing field.

However, the included studies were generally rated to be of a medium (*N* = 9) quality, with only two studies meeting all of the CASP (Critical Appraisal Skills Programme, 2024) criteria. Key methodological limitations included limited transparency around theoretical underpinnings, research design, researcher-participant relationships, data collection, and data analysis. These limitations may introduce bias or restrict the depth and reliability of the evidence synthesised here. Consequently, the themes and conclusions of this review should be interpreted cautiously, as they may reflect the limitations of the included studies rather than the phenomena themselves. Future research with more rigorous methodology is needed to confirm and extend these findings.

A strength of this review was its broad perspective across different stakeholders (people with dementia, caregivers, professionals) and therapy types, enabling a diverse exploration of perspectives, techniques and outcomes. However, while the inclusion of different stakeholders is a strength, it is also possible that different stakeholders prioritise different outcomes. For example, professionals may emphasise observable improvements in mood or functioning, whereas people with dementia may value feeling heard, understood, or socially connected. These differences highlight the need for therapy services to consider the lived experience of people with dementia alongside professional perspectives, to ensure interventions are meaningful.

Additionally, it was often unclear which specific elements of interventions participants were referring to within the studies which explored multicomponent interventions (Birtwell & Dubrow-Marshall, 2018; Skov et al., 2022), meaning that some potentially relevant data could not be included within the meta-synthesis.

A significant limitation of the existing literature is the inconsistent reporting of specific dementia diagnoses and standardised measures of cognitive impairment. Only six studies reported such data, which is critical given the high heterogeneity of dementia presentations and associated variations in cognitive impairment. Without this information, it is difficult to determine the efficacy or suitability of specific therapeutic techniques or adaptations for specific profiles under the broad umbrella of dementias. This limits the generalisability of findings and makes it difficult for clinicians to effectively target interventions. Future research must prioritise clear reporting of participant cognitive profiles.

It is also important to note that some of the findings may be specific to certain types of talking therapies, intervention contexts, or service settings. Consequently, these results may not generalise across all therapy types or settings, and care should be taken when extrapolating the findings.

As well as this, whilst the authors ensured that no first-order or second-order data was duplicated in the synthesis process, we also acknowledge the potential for over-representation of findings given that two of the included UK studies (Griffiths et al., 2021; Sass et al., 2022) and two of the included Swedish studies (Blomberg et al., 2024; Svedin et al., 2023) drew from the same intervention contexts and participant populations.

Additionally, individuals from ethnically diverse backgrounds are consistently underrepresented in dementia research (Brijnath et al., 2022) and the solely European studies focussed on primarily white participants in this review are no exception. The lack of diversity may have skewed the themes and conclusions identified here, as the experiences, adaptations, and outcomes of underrepresented groups are largely absent. Future research should prioritise inclusivity to ensure that findings apply to diverse groups.

## Conclusion

This systematic review and meta-synthesis provides an up-to-date overview on the experiences of people with dementia in accessing and engaging in talking therapies. Talking therapies can have a positive effect on individuals’ ability to address, process, and accept difficult life situations, however, require several adjustments to be made in order to maximise benefits. This includes changes in service funding, therapy structure, therapy impact measurement, and dementia-specific training. Additionally, involving people with dementia, caregivers and dementia professionals in therapy planning and delivery can also improve outcomes. Further research should explore the efficacy and suitability of specific elements of talking therapies, with the aim of developing tailored interventions for this population. It is also crucial to explore the level of access to talking therapies for people with dementia from ethnically diverse backgrounds to ensure inclusivity and address potential disparities in service provision.

## Supplemental Material


Supplemental Material - The Experience of People With Dementia in Accessing and Engaging in Talking Therapies for Mental Health Difficulties: A Systematic Review and Thematic Meta-Synthesis
Supplemental Material for The Experience of People With Dementia in Accessing and Engaging in Talking Therapies for Mental Health Difficulties: A Systematic Review and Thematic Meta-Synthesis by David Zammitt, Caroline Fearn, Amber John, Madalena Lykourgos, Suman Kurana, Ollie Hayes, and Joshua Stott in Dementia.

## Data Availability

Data that support the findings of this study are available from the corresponding author, upon reasonable request.*
