# Supplementary material for: The Experience of People With Dementia in Accessing and Engaging in Talking Therapies for Mental Health Difficulties: A Systematic Review and Thematic Meta-Synthesis
Source: Dementia (London). 2025 Dec 26;25(6):1448–74. doi: 10.1177/14713012251408694 (PMC13304917; doi:10.1177/14713012251408694)
Supplement: Supplemental Material - The Experience of People With Dementia in Accessing and Engaging in Talking Therapies for Mental Health Difficulties: A Systematic Review and Thematic Meta-Synthesis [file sj-pdf-1-dem-10.1177_14713012251408694.pdf]

Appendix A

Systematic Search Strategy

Searches of three electronic databases were conducted in February 2024 across two the PsycINFO and CINAHL databases within the Ovid interface, as well as directly accessing the Web of Science database. Reference lists within all studies which were included for full text screening were also searched for additional relevant literature.

PsycINFO search

1 exp dementia/

2 (dementia* or Alzheimer* or Huntington* or Lewy Bod* or Parkinson*).ti,ab.

3 (mental health adj2 (therap* or psychotherap*)).ti,ab.

4 (mental illness adj2 (therap* or psychotherap*)).ti,ab.

5 (psychopathology adj2 (therap* or psychotherap*)).ti,ab.

6 (psychiatric adj2 (therap* or psychotherap*)).ti,ab.

7 (psycholog* adj2 (therap*)).ti,ab.

8 mindful*.ti,ab.

9 (IAPT or improving access to psychological therapy or improving access to psychological therapies).ti,ab.

10 "acceptance and commitment therapy".ti,ab.

11 psychotherapeutic counseling/

12 counseling/ or community counseling/ or counselling.ti,ab.

13 exp Cognitive Behavior Therapy/ or ("cognitive behavio?r therapy" or "CBT").ti,ab.

14 "guided self help".ti,ab.

15 "behavio?ral activation".ti,ab.

16 ("couple therapy" or "couples therapy").ti,ab.

17 psychodynamic.ti,ab.

18 psychoanalysis.ti,ab.

19 (EMDR or "eye movement desensitisation and reprocessing").ti,ab.

20 (DBT or "dialectical behavio?r therapy").ti,ab.

21 (REBT or "rational emotive behavio?r therapy").ti,ab.

22 "interpersonal therapy".ti,ab.

23 ("compassion focused therapy" or "compassion-focused therapy").ti,ab.

24 ("talking therapy" or "talking therapies").ti,ab.

25 1 or 2

26 3 or 4 or 5 or 6 or 7 or 8 or 9 or 10 or 11 or 12 or 13 or 14 or 15 or 16 or 17 or 18 or 19 or 20 or 21 or 22 or 23 or 24

27 25 and 26

CINAHL search

(TI (dementia* OR Alzheimer* OR Huntington* OR Lewy Bod* OR Parkinson*) OR AB (dementia* OR Alzheimer* OR Huntington* OR Lewy Bod* OR Parkinson*)) AND (TI (psychotherap* OR “psychological therap*” OR “guided self help” OR “behavioural activation” OR “behavioral activation” OR “couple therapy” OR “couples therapy” OR psychodynamic OR psychoanalysis OR EMDR OR “eye movement desensitisation and reprocessing” OR DBT OR “dialectical behaviour therapy” OR “dialectical behavior therapy” OR REBT OR “rational emotive behaviour therapy” OR “rational emotive behavior therapy” OR “interpersonal therapy” OR "compassion focused therapy" OR "compassion-focused therapy" OR “acceptance and commitment therapy” OR "cognitive behavioural therapy” OR “cognitive behavioral therapy” OR “cognitive behaviour therapy” OR “cognitive behavior therapy” OR CBT OR "talking therapy" OR “talking therapies” OR mindful* OR “compassion focused therapy” OR counselling OR counseling OR IAPT OR “improving access to psychological therapy” OR “improving access to psychological therapies”) OR AB (psychotherap* OR “psychological therapy” OR “guided self help” OR “behavioural activation” OR “behavioral activation” OR “couple therapy” OR “couples therapy” OR psychodynamic OR psychoanalysis OR EMDR OR “eye movement desensitisation and reprocessing” OR DBT OR “dialectical behaviour therapy” OR “dialectical behavior therapy” OR REBT OR “rational emotive behaviour therapy” OR “rational emotive behavior therapy” OR “interpersonal therapy” OR "compassion focused therapy" OR "compassion-focused therapy" OR “acceptance and commitment therapy” OR "cognitive behavioural therapy” OR “cognitive behavioral therapy” OR “cognitive behaviour therapy” OR “cognitive behavior therapy” OR CBT OR "talking therapy" OR “talking therapies” OR mindful* OR “compassion focused therapy” OR counselling OR counseling OR IAPT OR “improving access to psychological therapy” OR “improving access to psychological therapies”))

Web of Science search

TI=(dementia* OR Alzheimer* OR Huntington* OR Lewy Bod* OR Parkinson*) OR AB=(dementia* OR Alzheimer* OR Huntington* OR Lewy Bod* OR Parkinson*)

AND

TI=(psychotherap*) OR AB=(psychotherap*) OR TI=(“acceptance and commitment therapy”) OR AB=(“acceptance and commitment therapy”) OR TI=("cognitive behavioural therapy" OR “cognitive behavioral therapy” OR “cognitive behaviour therapy” OR “cognitive behavior therapy” OR “CBT” OR “cognitive therapy”) OR AB=("cognitive behavioural therapy" OR “cognitive behavioral therapy” OR “cognitive behaviour therapy” OR “cognitive behavior therapy” OR “CBT” or “cognitive therapy”) OR TI=("talking therapy" OR “talking therapies”) OR AB=("talking therapy" OR “talking therapies”) OR TI=(mindful*) OR AB=(mindful*) OR TI=(“compassion focused therapy”) OR AB=(“compassion focused therapy”) OR TI=(counselling OR counseling) OR AB=(counselling OR counseling) OR TI=(IAPT OR “improving access to psychological therapy” OR “improving access to psychological therapies”) OR AB=(IAPT OR “improving access to psychological therapy” OR “improving access to psychological therapies”) OR TI=(psychological therap*) OR AB=(psychological therap*) OR TI=(guided self help) OR AB=(guided self help) OR TI=(couple therapy) OR AB=(couples therapy) OR TI=(psychodynamic) OR AB=(psychodynamic) OR TI=(psychoanalysis) OR AB=(psychoanalysis) OR TI=(EMDR OR “eye movement desensitisation and reprocessing”) OR AB=(EMDR OR “eye movement desensitisation and reprocessing”) OR TI=(DBT OR “dialectical behaviour therapy” OR “dialectical behavior therapy”) OR AB=(DBT OR “dialectical behaviour therapy” OR “dialectical behavior therapy”) OR TI=(REBT OR “rational emotive behaviour therapy”) OR AB=(REBT OR “rational emotive behaviour therapy”) OR TI=(“interpersonal therapy”) OR AB=(“interpersonal therapy”) OR TI=("compassion focused therapy" OR "compassion-focused therapy") OR

AB=("compassion focused therapy" OR "compassion-focused therapy") OR TI=(“behavioural activation” OR “behavioral activation”) OR AB=(“behavioural activation” OR “behavioral activation”)

Appendix B

Quality Assessment Criteria

1. Was there a clear statement of the aims of the research?

2. Is a qualitative methodology appropriate?

3. Was the research design appropriate to address the aims of the research?

4. Was the recruitment strategy appropriate to the aims of the research?

5. Was the data collected in a way that addressed the research issue?

6. Has the relationship between researcher and participants been adequately considered?

7. Have ethical issues been taken into consideration?

8. Was the data analysis sufficiently rigorous?

9. Is there a clear statement of findings?

10. How valuable is the research?

Additional item (Long, French & Brooks, 2020): Are the study's theoretical underpinnings (e.g. ontological and epistemological assumptions; guiding theoretical framework(s)) clear, consistent and conceptually coherent?
